# Integrated metabolomics and mass spectrometry imaging reveal the Anti-Alzheimer’s mechanisms of turmeric and curcumin

**DOI:** 10.3389/fcell.2026.1895521

**Published:** 2026-08-18

**Authors:** Zehua Yang, Yanyan Zeng, Jiahui Deng, Shuiping Zhang, Jing Xue, Qiaohui Li, Yi Feng, Yu Zeng

**Affiliations:** 1 School of Chinese Materia Medica Guangdong Pharmaceutical University Guangzhou China, Guangzhou, Guangdong, China; 2 Hospital Pharmaceutical Preparation R&D Lab, Guangdong Provincial Hospital of Chinese Medicine, Guangzhou, Guangdong, China

**Keywords:** AFAI-MSI, Aβ1-42, curcumin, metabolomics, turmeric residue

## Abstract

**Introduction:**

*Turmeric* and its major bioactive component, curcumin, have shown promising potential in the prevention and management of Alzheimer’s disease (AD). However, the underlying molecular mechanisms and the contribution of turmeric residues remain incompletely understood. This study aimed to investigate the protective effects of curcumin and turmeric residue in an Aβ1‐42‐induced AD mouse model and to elucidate their metabolic regulatory mechanisms.

**Methods:**

The phytochemical profiles of turmeric and turmeric residue were characterized using UPLC–MS/MS combined with bioinformatics analysis. An Aβ1‐42‐induced AD mouse model was established, and mass spectrometry imaging (AFAI‐MSI) was applied to determine the optimal modeling time point. The therapeutic effects of curcumin and turmeric residue were evaluated through behavioral assessments, including the Morris water maze test, as well as histopathological and molecular analyses involving H&E staining, Nissl staining, immunofluorescence, and immunohistochemistry. Furthermore, non‐targeted metabolomics was performed to identify metabolic alterations associated with treatment.

**Results:**

UPLC ‐MS/MS analysis identified 132 and 104 compounds in turmeric and turmeric residue, respectively, with 10 compounds showing significant differences in abundance between the two materials. AFAI‐MSI analysis indicated that three weeks after Aβ1‐42 injection represented an optimal time point for AD model establishment. Behavioral experiments demonstrated that both curcumin and turmeric residue significantly improved spatial learning and memory impairment in AD mice. Histological and molecular analyses revealed reduced neuronal damage and modulation of Aβ1‐42 and Iba‐1 expression in brain tissues. Metabolomic profiling identified 25 differential plasma metabolites and 38 differential brain metabolites following treatment. Pathway enrichment analysis suggested that these metabolic alterations were mainly associated with ether lipid, sphingolipid, glycerophospholipid, glutamate, purine, and arachidonic acid metabolism.

**Discussion:**

These findings demonstrate that curcumin and turmeric residue exert neuroprotective effects against AD‐related pathological alterations by regulating metabolic disturbances and restoring metabolite homeostasis. The integration of chemical profiling, AFAI‐MSI, and metabolomics provides new insights into the therapeutic potential and metabolic mechanisms of turmeric‐derived resources in Alzheimer’s disease.

## Graphical Abstract

**Figure:**
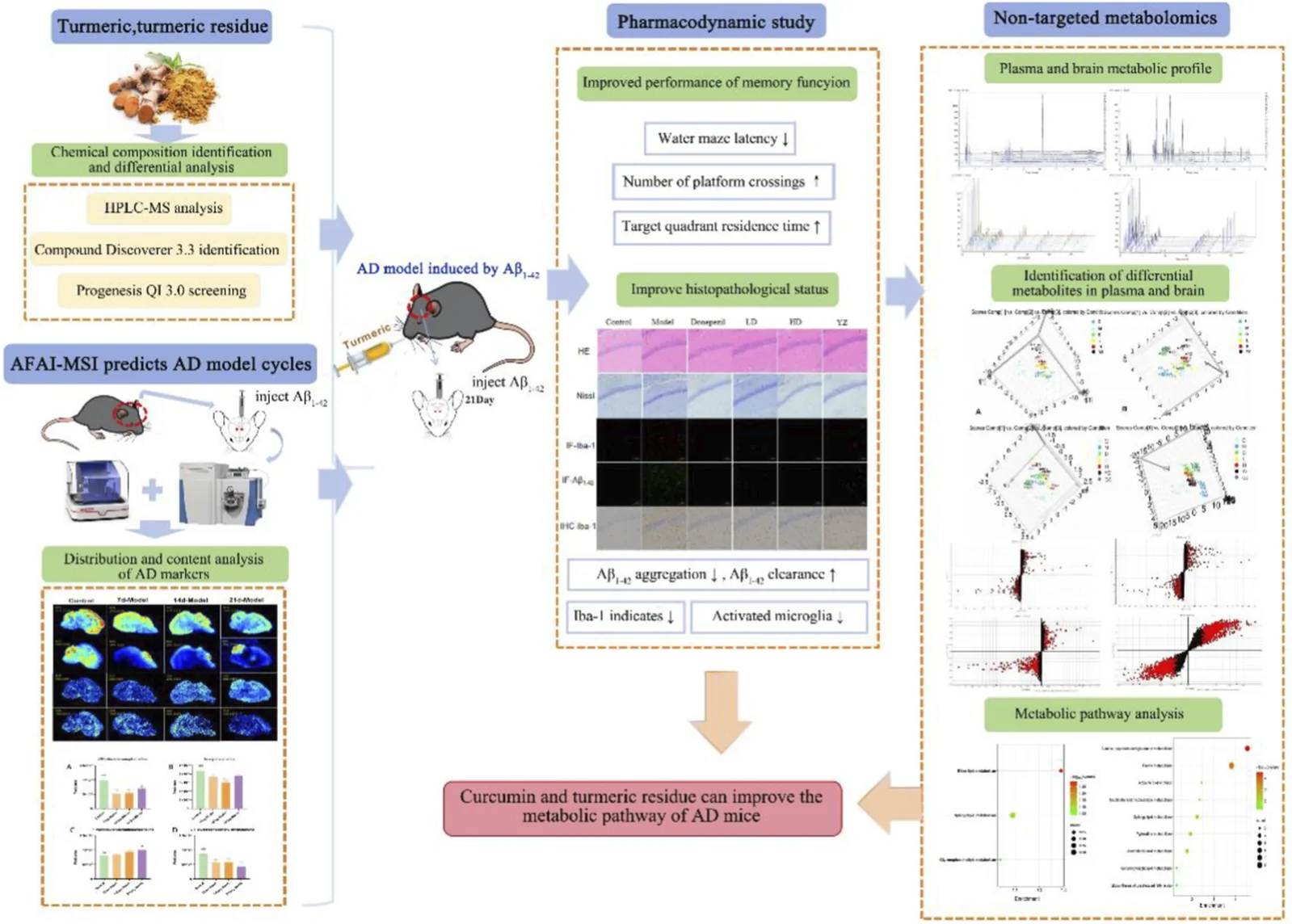

## 1 Introduction

Alzheimer’s disease is a neurodegenerative disorder. It is marked by the accumulation of extracellular senile plaques, intracellular neurofibrillary tangles within brain cells, and abnormal synaptic function of neurons (Kamatham et al., 2024). This results in a considerable loss of neurons and is clinically shown as memory impairment, aphasia, agnosia, visuospatial skill problems, executive dysfunction, and changes in personality and behavior. The main pathological characteristics are β-amyloid (Aβ) plaques and hyperphosphorylated tau (pTau) protein tangles (Ye et al., 2024). The aggregation of Aβ forms deposits, triggers neuroinflammation, and hinders intercellular communication. Meanwhile, the phosphorylation of the Tau protein forms neurofibrillary tangles, disrupting the exchange of neuronal materials. Among the various pathogenic mechanisms, the most widely accepted amyloid hypothesis proposes that Aβ aggregates and deposits into plaques, speeding up processes such as neuroinflammation, neuronal damage, and synaptic loss. These pathological processes further accelerate Aβ aggregation, creating a cascade amplification effect that leads to neural damage and eventually causes Alzheimer’s disease.

Increasing evidence suggests that food-medicine homologous natural products possess significant neuroprotective properties and may represent promising candidates for the prevention and treatment of neurodegenerative disorders (Ye et al., 2025) *Turmeric*’s primary active compound is curcumin, a polyphenolic substance known for its anti-inflammatory properties (Tagde et al., 2021). Curcuminoids and sesquiterpenoids have been identified as the principal bioactive constituents of Curcuma longa, contributing to its anti-inflammatory and neuroprotective activities (Dong et al., 2025). These properties have been extensively investigated and applied in the treatment of various diseases, including dermatological disorders and anti-cancer therapies (Hsu et al., 2023; Zhong et al., 2025). In the context of AD, modern medical research indicates that turmeric may have therapeutic benefits (Lamichhane et al., 2024; Reddy et al., 2018). Turmeric’s diverse properties have been shown to mitigate AD symptoms by reducing β-amyloid plaques (Kaddurah-Daouk et al., 2013),exerting anti-inflammatory (Tsuchida et al., 2020) and antioxidant effects (Ayati et al., 2019), and decreasing microglia formation (Yuan et al., 2016). These benefits contribute to improved memory and cognitive function in AD patients.

Recent advancements in mass spectrometry imaging (AFAI-MSI) have established it as a revolutionary molecular imaging modality (Li et al., 2024). Recent advances in spatial metabolomics have enabled visualization of metabolite distribution within tissues, providing novel insights into disease-associated metabolic reprogramming and the spatial heterogeneity of biological processes (Zhu, 2025). Among its variants, ambient flow-assisted ionization AFAI-MSI has garnered significant attention in the biomedical field due to its distinct advantages, including the elimination of prerequisite sample pretreatment, high sensitivity, and extensive metabolite coverage. The objectives of the present study were as follows: (1) to establish an optimized Aβ_1-42_-induced AD mouse model using MSI, enabling the investigation of the spatiotemporal progression of metabolic perturbations in critical brain regions over time; (2) to evaluate the therapeutic efficacy of curcumin and curcuminoid-derived residues on cognitive function, Aβ deposition, and neuroinflammation; (3) to elucidate the neuroprotective mechanisms of these compounds by MSI with global plasma and brain metabolomics, identifying key pathways such as glycerophospholipid metabolism and sphingolipid metabolism.

Our findings not only validate the potential of curcuminoid-derived residues as a sustainable source of neuroprotective compounds but also provide novel mechanistic insights into how curcuminoids modulate region-specific metabolic networks in Alzheimer’s disease. This research underscores the powerful role of spatial metabolomics in bridging the gap between traditional herbal medicine and modern precision medicine for neurodegenerative diseases.

## 2 Materials and methods

### 2.1 Materials and reagents

The study utilized the following chemicals and reagents:

The *turmeric* dregs were provided by Guangdong Yifang Pharmaceutical Co., Ltd. Aβ_1-42_ (bs-0107P) was purchased from Beijing Biosynthesis Biotechnology Co., Ltd. Curcumin (purity >98.9%), demethoxycurcumin (purity >98.5%), and bisdemethoxycurcumin (purity >95%) were purchased from the China National Center for Food and Drug Control. Tetrahydrocurcumin (purity >98%) was obtained from Shanghai Yuanye Bio-Technology Co., Ltd. Donepezil hydrochloride tablets were obtained from Eisai (China) Pharmaceutical Co., Ltd. Anti-Iba-1 Rabbit pAb (GB113502)was obtained from Shanghai Pudong Jinhuan Medical Products Co., Ltd. he staining kit (G1003), toluidine blue staining solution (G1036), and immunohistochemistry kit with DAB chromogen (G1212) were obtained from Wuhan Servicebio Technology Co., Ltd. All other reagents and chemicals were of analytical grade.

### 2.2 Sample preparation for UHPLC-Q/TOF-MS analysis

The crude *turmeric* medicinal material and *turmeric* residue were pulverized using a high-speed grinder. Approximately 1 g of powder from three different batches (passing through an 80-mesh sieve) was accurately weighed and soaked in 5 mL of 100% methanol for 2 h. The mixture was then sonicated for 15 min and centrifuged at 13,000 rpm for 5 min. The supernatant was collected. The residue was re-extracted sequentially with 5 mL of 80% methanol and 5 mL of 60% methanol, repeating the sonication and centrifugation steps. The combined supernatants were diluted with 80% methanol to a final concentration of 4 mg/mL and filtered through a 0.22 μm organic microporous membrane to obtain the test solution.

The curcumin residue solution was prepared according to established methods (Seo et al., 2011; Hossen et al., 2017). Briefly, the residue powder (passing through an 80-mesh sieve) was mixed with a 75% ethanol solution at a ratio of 1:10 (w/v) and shaken on a rotary shaker for 3 days. The resulting solution was filtered successively through cheesecloth and filter paper to obtain the final extract.

Blood samples were collected and centrifuged to obtain serum. For metabolomic analysis, each serum sample (200 μL) was subjected to protein precipitation by mixing with a 1:1 (v/v) mixture of acetonitrile and methanol. The mixture was vortexed and centrifuged at low speed, and the supernatant was collected for analysis.

Brain tissues were homogenized in a pre-cooled extraction solution of methanol, acetonitrile, and water (2:2:1, v/v/v). After centrifugation, the supernatant was collected, and the extraction procedure was repeated twice. The combined supernatants were evaporated to dryness under a gentle nitrogen stream. The residue was reconstituted in 80% methanol, followed by brief vortexing and centrifugation. The final supernatant was transferred for LC-MS analysis.

For quality control, an equal volume (10 μL) of the prepared supernatant from each plasma and brain tissue sample was pooled to create a composite QC sample. This QC sample was injected at regular intervals throughout the analytical sequence to monitor the stability and reproducibility of the instrument system.

### 2.3 Composition analysis by UPLC- MS/MS

The sample solution was filtered through a 0.22 μm organic microporous membrane prior to analysis. Liquid chromatography-tandem mass spectrometry (LC-MS/MS) was performed using a Thermo Scientific Q Exactive Orbitrap™ MS system (Thermo Fisher Scientific, USA). Chromatographic separation was achieved on an ACQUITY UPLC HSS T3 C18 column (2.1 mm × 100 mm, 1.8 μm) maintained at 40 °C. The mobile phase consisted of (A) 0.1% formic acid in water and (B) 0.1% formic acid in acetonitrile, delivered at a flow rate of 0.3 mL/min with the following gradient program: 0–10 min, 80%–65% A; 10–30 min, 65%–45% A; 30–38 min, 45%–2% A; 38–41 min, 2% A; 41–41.1 min, 2%–60% A; 41.1–46 min, 60% A. The injection volume was 1 μL.

Mass spectrometry was conducted in both positive and negative electrospray ionization (ESI) modes with a full-scan range of m/z 70–1050. Key MS parameters were set as follows: sheath gas flow, 40 arb; auxiliary gas flow, 10 arb; heater temperature, 350 °C; capillary temperature, 320 °C; spray voltage, ±3500 V; S-lens RF level, 50 V; Full Scan, 70,000; ddMS2-Discovery, 17,500.

Raw data were processed using Progenesis QI 3.0 and Compound Discoverer 3.3 software for baseline correction, peak picking, retention time alignment, and intensity integration. Preliminary compound identification was carried out in Compound Discoverer 3.3, followed by multivariate statistical analysis in Progenesis QI 3.0. Principal component analysis (PCA) and orthogonal partial least squares-discriminant analysis (OPLS-DA) were performed using EZinfo 2.0 (Anmol et al., 2024). Differential compounds were screened with variable importance in projection (VIP) > 2.0 and ANOVA p < 0.05. Final structural identification was accomplished by matching accurate mass and MS/MS spectra against the Human Metabolome Database (HMDB), mzCloud, PubChem, ChemSpider, and OTCML.

### 2.4 Animal and experimental groups

Sixty SPF-grade, 2-month-old male C57BL/6 mice, weighing between 18 and 22 g, were purchased from Guangdong Zhiyuan Biotechnology Co., Ltd. The animal production license number is SCXK (yue) 2021-0057, and the experimental animal quality certificate number is No. 44826500004898. The experimental animals were kept in the SPF- level laboratory of the Animal Experiment Center of Guangdong Pharmaceutical University. The unit use license number is SYXK (Guangdong) 2017- 0125, and the experiment number is SPF2022398. The ambient temperature is 22 °C–26 °C, and the relative humidity is 50%–60%. This experiment strictly complied with the relevant regulations on animal welfare ethics and protection in Guangdong Province. All animals were subjected to 7 days of acclimatization, during which time the rats had free access to food and water.

SPF-grade 2-month-old male C57BL/6 mice, 24 in total, weighing 18–22 g each, were purchased from the Guangdong Medical Experimental Animal Center with license number SYXK (Guangdong) 2023-0015. All animals were housed at the Animal Center of Guangdong Pharmaceutical University with license number SYXK (Guangdong) 2022-0125. They were provided with *ad libitum* access to food and water, maintained in an environment with a temperature of 20 °C–25 °C, humidity of 40%–70%, and a 12-h light/dark cycle. After a 7-day acclimation period, the mice were randomly assigned to four groups: the Sham Surgery Group (n = 6), the Modeling for 7 Days Group (n = 6), the Modeling for 14 Days Group (n = 6), and the Modeling for 21 Days Group (n = 6). The sham-operated group received bilateral hippocampal injections of sterile saline, while the other mouse groups received bilateral hippocampal injections of Aβ_1-42_ (410 pmol/μL).

The 60 C57BL/6 mice were randomly divided into a sham-operated group (0.1 mL/g distilled water, Control), a modeling group (0.1 mL/10 g distilled water, Model), a donepezil group (0.76 mg/kg donepezil hydrochloride solution, Donepezil), a *turmeric* residue group (1.2 g (raw drug)/kg, YZ), a low-dose curcumin group (10 mg/kg, LD), and a high-dose curcumin group (20 mg/kg, HD), with 10 mice in each group. Each mouse is first given gastric gavage for a week, and then brain-localized injections are performed on the eighth day of the experiment, with a 2-day recovery period for the mice; on the 10th day of the experiment, gavage is repeated and continued for 3 weeks. The sham-operated group received bilateral hippocampal injections of sterile saline, while the other mouse groups received bilateral hippocampal injections of Aβ_1-42_ (410 pmol/μL).

### 2.5 AFAI-MS imaging establishes

Before modeling, the mice were fasted for 12 h with free access to water. Anesthesia was induced by intraperitoneal injection of 1% pentobarbital sodium (0.08 mL/10 g). A microsyringe was used to bilaterally inject 1.5 μL of Aβ_1-42_ solution into the hippocampal CA1 region (stereotaxic coordinates: AP: −0.22 cm, ML: ±0.2 cm, DV: −0.2 cm) (affINRA-INSERM et al., 2003). Each injection was performed over 10 min, and the needle was left in place for an additional 15 min before withdrawal. The same procedure was repeated on the contralateral side. Post-surgery, the incision was sutured and disinfected, and mice were placed on a sterile heating pad until full recovery.

Mass spectrometry imaging was performed using an ambient liquid extraction-based setup (AFAI-MSI system, Vecto (Beijing) Technology Co., Ltd.). The spray solvent was methanol: water (8:2, V/V) as the spray solvent in both positive and negative ion modes, with a constant flow rate of 5 μL/min. High-resolution mass spectrometry data were acquired using a Q Exactive Focus instrument (Thermo Fisher Scientific, USA) with the following parameters: spray voltage, ±7000 V; capillary temperature, 350 °C; full MS scan mode with a mass range of 100–1000 Da; and resolution set at 70,000. Sheath gas and auxiliary gas were turned off, while the extraction gas flow was maintained at 45 L/min. The spray gas pressure was controlled between 0.6 and 0.8 MPa, and the geometric alignment included a sprayer-to-surface distance of 0.7 mm, spray-to-guide tube distance of 3 mm, and orifice-to-guide tube distance of 10 mm.

### 2.6 Behavioral experiments

The spatial navigation assessment was conducted over 5 days (days 16–20). Mice were placed in one of four pool quadrants facing the water. Escape latency was the time to identify the submerged platform (max 90s). Platform identification (stay >10s) defined escape latency. Mice not finding it within 90s were guided to it and left for 10s. This was repeated over 5 days.

On day 6, the platform was removed for behavioral testing. Mice were placed opposite the original platform location. Crossings of the former platform location and time in the target quadrant were recorded following standard protocols.

### 2.7 Tissue collection and processing

Following successful model establishment, mice were anesthetized with isoflurane and euthanized by cervical dislocation at designated time points. Blood was collected in heparinized tubes and centrifuged to obtain plasma. The whole brain was rapidly excised. One hemisphere was snap-frozen in liquid nitrogen and stored at −80 °C for subsequent metabolomic analysis. Sagittal sections (12 μm thickness) encompassing the hippocampal CA1 injection site were prepared using a Leica CM1860 cryostat. For each sample, six consecutive sections were allocated: three for spatial metabolomics imaging, two for conventional homogenate-based untargeted metabolomics, and one for histological evaluation.

### 2.8 Histopathological and immunohistochemical staining

The contralateral hemisphere was fixed in 4% paraformaldehyde for 24 h, followed by standard dehydration, clearing, and paraffin embedding. Coronal sections (6 μm thickness) were prepared for subsequent staining.

Hematoxylin and eosin (H&E) staining was conducted. Deparaffinized sections were stained following standard H&E protocols to assess general tissue morphology and neuronal structure.

Nissl staining was performed. Sections were stained with toluidine blue to visualize Nissl bodies in neuronal cytoplasm. Differentiation was monitored under a microscope prior to dehydration, clearing, and mounting.

Immunohistochemistry (IHC) for Iba1 was conducted. After deparaffinization and antigen retrieval, endogenous peroxidase was quenched with 3% H_2_O_2_. Sections were blocked with 3% BSA and incubated overnight at 4 °C with a rabbit anti-Iba1 primary antibody (1:1000 dilution) (Bhat et al., 2015). This was followed by incubation with a horseradish peroxidase (HRP)-conjugated goat anti-rabbit secondary antibody. Signal was developed using 3,3′-diaminobenzidine (DAB), and sections were counterstained with hematoxylin.

Immunofluorescence (IF) staining for Iba1 and Aβ1-42 was performed. A tyramide signal amplification (TSA)-based multiplex immunofluorescence protocol was employed. After blocking, sections were sequentially incubated with primary antibodies against Iba1 and Aβ1-42, each followed by their respective HRP-conjugated secondary antibodies and TSA fluorophore treatment, with a microwave heat treatment step between each round to denature antibodies (Bhat et al., 2015). Nuclei were counterstained with DAPI.

### 2.9 Image acquisition and analysis

Stained sections were imaged using a light microscope (H&E, Nissl, IHC) or a fluorescence microscope (IF). For IHC, Iba1-positive area and cell density were quantified using ImageJ software.

### 2.10 Non-targeted MS measurements

Non-targeted metabolomic analysis was performed using an ultra-performance liquid chromatography system (Ultimate 3000, Thermo Scientific) coupled to a high-resolution mass spectrometer (Q Exactive Focus, Thermo Scientific). Sample preparation involved centrifugation using a refrigerated high-speed centrifuge. Chromatographic separation was achieved on a Waters ACQUITY UPLC HSS T3 C18 column (2.1 mm × 100 mm, 1.8 μm) maintained at 40 °C, with an autosampler temperature of 8 °C and a flow rate of 0.3 mL/min.

For plasma analysis, the mobile phase consisted of 0.1% formic acid in water (A) and 0.1% formic acid in acetonitrile (B) (Vasco et al., 2024). The gradient elution program was: 0–2 min, 98% A; 2–5 min, 98%–65% A; 5–13 min, 65%–0% A; 13–15 min, 0% A; 15–15.1 min, 0%–98% A; 15.1–18 min, 98% A. The injection volume was 1 μL.

For brain tissue analysis, the mobile phase consisted of 2 mM ammonium formate and 0.1% formic acid in water (A) and acetonitrile (B). The gradient elution program was: 0–1 min, 98% A; 1–2.5 min, 98%–78% A; 2.5–18 min, 78%–25% A; 18–24 min, 25%–0% A; 24–26 min, 0% A; 26–26.1 min, 0%–98% A; 26.1–28 min, 98% A. The injection volume was 2 μL.

Mass spectrometry was conducted on a Q Exactive Focus instrument (Thermo Scientific) in both positive and negative electrospray ionization (ESI) modes, with a full-scan range of m/z 70–1050. Key parameters were set as follows: sheath gas flow rate, 40 arb; auxiliary gas flow rate, 10 arb; heater temperature, 350 °C; capillary temperature, 320 °C; spray voltage, +3500 V (positive) and −3200 V (negative); S-lens RF level, 50 V. Full MS scans were acquired at a resolution of 70,000, and data-dependent MS^2^ scans were triggered at a resolution of 17,500 using stepped normalized collision energies of 20, 30, and 40 eV.

The raw data were processed and normalized using Progenesis QI 3.0 and Compound Discoverer 3.3 to obtain a peak intensity matrix. Multivariate analyses, including PCA and OPLS-DA, were performed to assess metabolic profiles and identify differential metabolites. Metabolites with a VIP score ≥1, fold change >2, and ANOVA p-value <0.05 were selected for identification via HMDB, mzCloud, and PubChem. Significant metabolic pathways were subsequently identified through enrichment analysis in MetaboAnalyst 5.0 (p < 0.05, impact >0).

### 2.11 Statistical analysis

The analysis was performed using GraphPad Prism 9.4 software, with all data expressed as mean ± standard deviation (x̄ ± s); in the Morris water maze experiment, the escape latency was analyzed using a two-way ANOVA (two-way ANOVA), and other experimental groups were analyzed using one-way ANOVA for intergroup comparisons, with multiple comparisons performed using the Turkey method. A *p* < 0.05 was considered to indicate a significant difference between groups.

## 3 Results

### 3.1 The 3-week model best mimics AD as characterized by AFAI-MS

**FIGURE 1:**
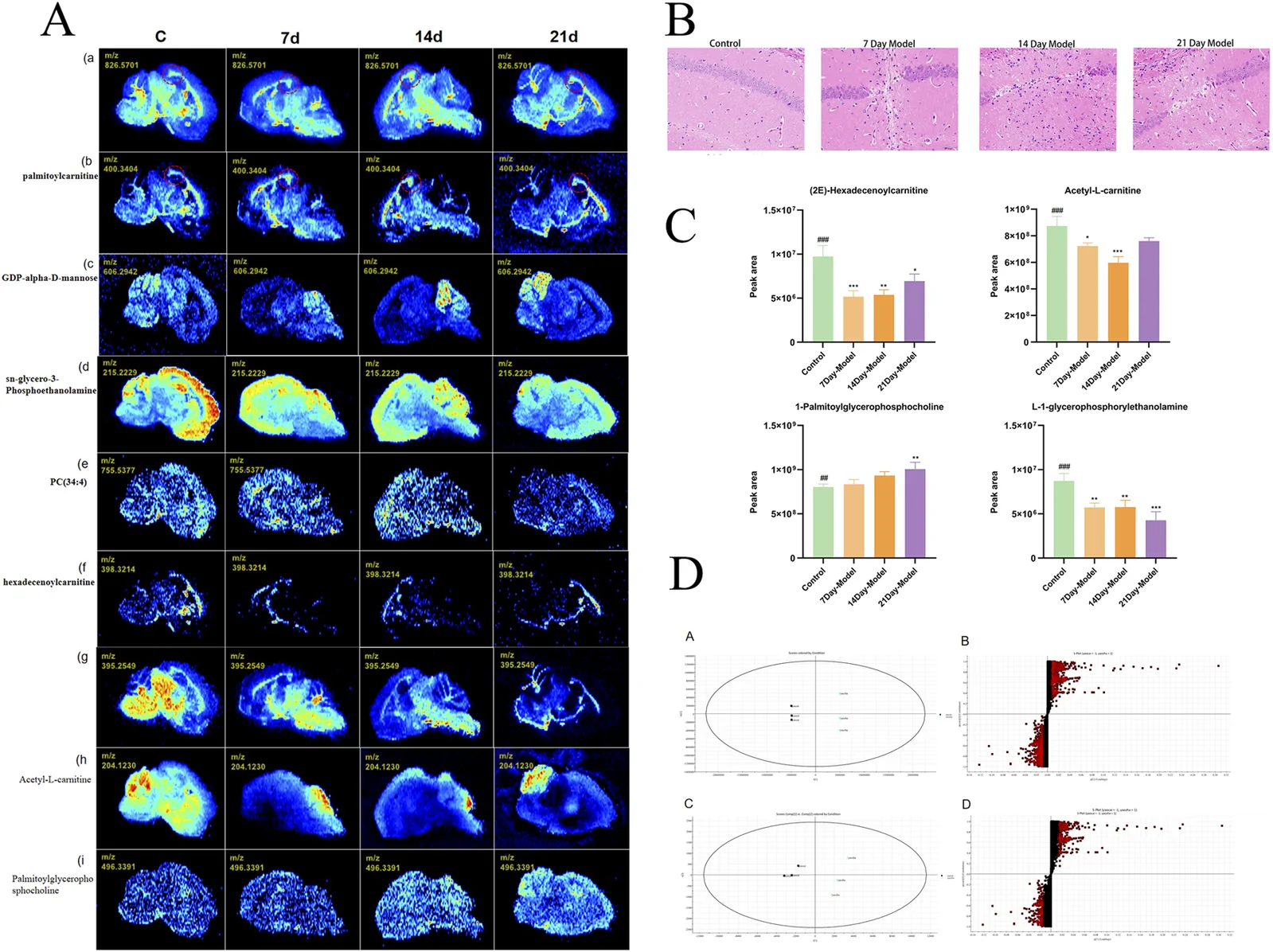
Mass spectrometry (MS) data analysis. **(A)** Spatial mass spectrometry image. **(B)** Aβ_1-42_ induces pathological changes. **(C)** Results of relative content of whole brain markers in Control, 7-day Model, 14-day Model, and 21-day Model mice (n = 3). Note: *: p < 0.05, **: p < 0.01, ***: p < 0.001, compared with sham operation group. **(D)** Orthogonal partial least squares-discriminant analysis (OPLS-DA) of metabolic profiles between Curcuma and residue groups.

The most common metabolites in brain tissue, within the mass range of m/z 100 to 1000, include lipid metabolism and energy metabolism. Here, we first report on the mass spectrometry profiles that distinguish Alzheimer’s disease-affected tissue from normal tissue. Figure 1A shows brain tissue slices from the sham surgery group, 7 days after modeling, 14 days after modeling, and 21 days after modeling, along with their corresponding optical images.

The results show that in AD mice, lipid metabolism abnormalities lead to a decrease in the expression of phosphatidyl cholines (PC 34:4, m/z 755.5377) (Figure 1Ae), phosphocholine, and glycero-Phosphoethanolamine (m/z 215.2229) (Figure 1Ad). This is consistent with findings from previous literature (Rahmani et al., 2016). Specifically, after one and 2 weeks of localized injection with Aβ1-42, the abundance of PC (34:4) gradually decreased, and a sharp decrease was observed in the third week, suggesting that with the extension of the modeling period in the Aβ1-42 injection group, the severity of lipid metabolic disorders deepens.

Biomarkers related to energy metabolism, such as palmitoylcarnitine (CAR, m/z 400.3404) (Figure 1Ab), showed an increase in abundance in the hippocampal region of mice in the Aβ1-42 injection group. Hexadecenoylcarnitine (m/z 398.3214) (Figure 1Af) and acetyl-L-carnitine (m/z 204.1230) (Figure 1Ah) were found to have a lower overall content compared to the sham surgery group, suggesting that there is an abnormal energy metabolism in the brain of mice in the Aβ1-42 injection group (Zhang et al., 2013).

The overall content of the pro-inflammatory lysophosphatidylcholine (P-almitoylglycerophosphocholine, m/z 496.3391) (Figure 1Ai) gradually increased with the extension of the period in the Aβ_1-42_ injection group, with the hippocampal and cerebellar regions being the most significant. In an Alzheimer’s disease model, gut microbiota-derived lysophosphatidylcholine (LPC) exerts anti-neuroinflammatory and neuroprotective effects by activating the GPR119 receptor and subsequently inhibiting ACSL4-mediated ferroptosis (Zha et al., 2025).

Observations from histopathological staining results revealed that all three model groups exhibited varying degrees of tissue damage compared to the sham surgery group, with signs of cellular atrophy, pyknotic nucleoli, and darkened nuclear and cytoplasmic colors (Figure 1). The results of spatial mass spectrometry metabolomics showed that in the mouse brain regions 3 weeks after Aβ_1-42_ injection, the overall abundance of markers for abnormal lipid metabolism decreased most significantly compared to the sham surgery group (Figure 1). The overall abundance of markers related to inflammation increased most significantly compared to the sham surgery group (Figure 1C). Based on the distribution and changes in the abundance of these biomarkers, the metabolic disturbance in the brains of C57BL/6 mice 3 weeks after Aβ_1-42_ injection is more similar to that in AD mice.

### 3.2 The main components of curcumin and turmeric residue were identified by UHPL-MS/MS

The chemical constituents of *turmeric* crude drugs and processing residues were characterized using UPLC-QTOF-MS/MS. Based on high-resolution MS data, MS/MS fragmentation patterns, and literature comparisons, a total of 132 compounds were preliminarily identified in the crude *turmeric* material, while 105 compounds were detected in the residue. Among these, the crude material contained 37 sesquiterpenoids, 6 curcuminoids, 8 methoxy phenolic compounds, and 3 hydroxycinnamic acid derivatives, along with other active constituents. The residue was found to contain 35 sesquiterpenoids, 6 curcuminoids, 5 methoxy phenolic compounds, and 4 hydroxycinnamic acid derivatives, among others. The common components shared between the crude drug and its residue are summarized in Table 1.

**TABLE 1:** Identification of chemical components in *turmeric* and residue.

No.	Retention time/min	Compounds	Formula	Molecular weight	m/z	Mass error/ppm	References Ion
1	0.87	DL-Lysine	C6 H14 N2 O2	146.105	147.113	−1.35	[M + H]+1
2	0.88	DL-Arginine	C6 H14 N4 O2	174.111	175.119	−1.04	[M + H]+1
3	0.97	Asparagine	C4 H8 N2 O3	132.053	133.061	−1.59	[M + H]+1
4	0.98	L-pyroglutamic acid	C5 H7 N O3	129.042	130.050	−1.57	[M + H]+1
5	0.98	N-Acetylornithine	C7 H14 N2 O3	174.100	175.107	−1.33	[M + H]+1
6	1.03	Guanine	C5 H5 N5 O	151.049	152.057	−0.37	[M + H]+1
7	1.04	L-Tyrosine	C9 H11 N O3	181.074	182.081	−0.6	[M + H]+1
8	1.09	L-isoleucine	C6 H13 N O2	131.095	132.102	−0.85	[M + H]+1
9	1.09	6-Hydroxypicolinic acid	C6 H5 N O3	139.027	140.034	−0.83	[M + H]+1
10	1.36	Leucylproline	C11 H20 N2 O3	228.147	229.155	−0.71	[M + H]+1
11	1.52	Pyroquilon	C11 H11 N O	173.084	174.091	−0.99	[M + H]+1
12	1.63	2-piperidinobenzoic acid	C12 H15 N O2	205.110	206.117	−0.86	[M + H]+1
13	1.80	6-Methylquinoline	C10 H9 N	143.073	144.081	−0.45	[M + H]+1
14	1.88	Maltol	C6 H6 O3	126.032	127.039	−0.98	[M + H]+1
15	2.29	2-Pyridylacetic acid	C7 H7 N O2	137.048	138.055	−1.1	[M + H]+1
16	3.15	Metolachlor morpholinone	C14 H19 N O2	233.141	234.149	−1.09	[M + H]+1
17	3.33	4-Hydroxybenzaldehyde	C7 H6 O2	122.037	123.044	−0.97	[M + H]+1
18	3.37	3,4-Dimethylbenzoic acid	C9 H10 O2	150.068	151.075	−0.58	[M + H]+1
19	3.38	4-Isobutylbenzoic acid	C11 H14 O2	178.099	179.106	−0.68	[M + H]+1
20	3.47	4-Coumaric acid	C9 H8 O3	164.047	147.044	−0.8	[M + H-H2O]+1
21	3.87	Senkyunolide H	C12 H16 O4	224.105	207.101	−0.62	[M + H-H2O]+1
22	4.05	Isoferulic acid	C10 H10 O4	194.058	195.065	−0.62	[M + H]+1
23	4.49	2,4-Dimethylbenzaldehyde	C9 H10 O	134.073	135.080	−0.88	[M + H]+1
24	5.35	4-Phenylbutyric acid	C10 H12 O2	164.084	165.091	−0.47	[M + H]+1
25	5.38	4-Indolecarbaldehyde	C9 H7 N O	145.053	146.060	−0.83	[M + H]+1
26	5.79	Diphenyl carbonate	C13 H10 O3	214.063	215.070	−1.02	[M + H]+1
27	5.82	1-Naphthol	C10 H8 O	144.057	145.065	−1.08	[M + H]+1
28	6.31	Diethyl phosphate	C4 H11 O4 P	154.039	155.047	−1.38	[M + H]+1
29	6.47	Piceatannol	C14 H12 O4	244.073	245.081	−1.14	[M + H]+1
30	7.36	Veratrole	C8 H10 O2	138.068	139.075	−1.05	[M + H]+1
31	8.33	Carvone	C10 H14 O	150.104	151.112	−0.22	[M + H]+1
32	9.40	Dihydrokawain	C14 H16 O3	232.110	233.117	−1.26	[M + H]+1
33	12.70	Curcumin	C21 H20 O6	368.125	367.118	−1.55	[M + H]+1
34	12.74	Valethamate	C19 H31 N O2	305.235	306.242	−1.6	[M + H]+1
35	12.87	(±)-abscisic acid	C15 H20 O4	264.136	265.143	−1.21	[M + H]+1
36	16.09	Ferulic acid	C10 H10 O4	194.058	195.065	−0.65	[M + H]+1
37	17.15	Methyl cinnamate	C10 H10 O2	162.068	163.075	−0.8	[M + H]+1
38	17.48	Acetophenone	C8 H8 O	120.057	121.065	−1.04	[M + H]+1
49	18.50	Dihexylamine	C12 H27 N	185.214	186.221	−0.89	[M + H]+1
40	20.48	Hexadecanamide	C16 H33 N O	255.256	256.263	−1.49	[M + H]+1
41	20.78	L-(-)-carvone	C10 H14 O	150.104	151.112	−1.3	[M + H]+1
42	21.11	Flavanone	C15 H12 O2	224.083	225.091	−1.55	[M + H]+1
43	21.12	Bisdemethoxycurcumin	C19 H16 O4	308.104	309.112	−1.33	[M + H]+1
44	21.27	Polygodial	C15 H22 O2	234.162	235.169	−1.28	[M + H]+1
45	21.80	11-Ketotestosterone	C19 H26 O3	302.188	303.195	−1.61	[M + H]+1
46	21.88	Heptanophenone	C13 H18 O	190.136	191.143	−1.21	[M + H]+1
47	22.36	Demethoxycurcumin	C20 H18 O5	338.115	339.122	−1.34	[M + H]+1
48	23.26	trans-Cinnamaldehyde	C9 H8 O	132.057	133.065	−1.12	[M + H]+1
49	25.80	19-Nortestosterone	C18 H26 O2	274.193	275.200	−0.92	[M + H]+1
50	26.19	Benzyl methacrylate	C11 H12 O2	176.084	159.080	−0.98	[M + H-H2O]+1
51	26.47	(-)-Caryophyllene oxide	C15 H24 O	220.183	203.179	−0.77	[M + H-H2O]+1
52	27.64	α-Pyrrolidinovalerophenone	C15 H21 N O	231.162	232.169	−0.85	[M + H]+1
53	28.51	Curcumin	C21 H20 O6	368.126	369.133	−1.09	[M + H]+1
54	32.98	Zerumbone	C15 H22 O	218.167	219.174	−1.12	[M + H]+1

According to the results shown in Figure 1D, OPLS-DA and VIP analysis reveal that the medicinal herb group and the dregs group can be separated on either side of the central axis, with some differences in components between the two groups. By combining VIP (Variable importance in the projection) ≥ 2, Max fold change >2, and ANOVA P-value <0.05, 10 differential components were identified, including 1 curcuminoid compound, 3 sesquiterpenoid compounds, 2 flavonoid compounds, 2 monosaccharide compounds, 1 steroid compound, and 1 polyphenol compound. Among them, among the 6 batches of analytes at the same concentration, the average peak area of curcumol and curcumin oleoresin contained in 3 batches of medicinal dregs was higher than that of medicinal materials. Moreover, the total ion current chromatogram results of curcuma dregs indicated that the peak response value of enolic curcuminoids was higher than that of ketonic ones, suggesting that the medicinal dregs have potential development value.

### 3.3 Therapeutic effects of curcumin and turmeric residue on spatial memory impairment

As shown in Table 2, mice in the model group exhibited a significantly prolonged escape latency in the Morris water maze test compared to the control group. In the subsequent spatial probe trial (Figures 2A,C), the model group further demonstrated severe cognitive impairments, as indicated by a reduced number of platform crossings and decreased time spent in the target quadrant. These behavioral deficits are consistent with established Alzheimer’s disease phenotypes. Importantly, treatment with donepezil, curcumin, and *turmeric* residue significantly reversed these deficits, leading to shortened escape latency, increased platform crossings, and more time spent in the target quadrant. An increase in locomotor speed was also noted in the treatment groups.

**TABLE 2:** Escape latency in place navigation test (x̅ ± s,n = 6).

Group	Day1	Day2	Day3	Day4	Day5
Control	78.5 ± 11.41	73.9 ± 16.03	39.2 ± 16.66	31.3 ± 8.29	29.5 ± 11.24
Model	90.0 ± 0.00	90.0 ± 0.04	90.0 ± 1.03^####^	90.0 ± 5.01^####^	90.0 ± 5.58^####^
Donepezil	87.1 ± 2.87	62.6 ± 4.73^**^	27.3 ± 10.12^****^	23.1 ± 5.559^****^	22.5 ± 8.07^****^
LD	84.7 ± 8.13	68.5 ± 20.37	41.9 ± 6.08^****^	33.9 ± 8.91^****^	27.6 ± 6.96^****^
HD	74.9 ± 10.05	51.9 ± 2.18^****^	31.0 ± 5.02^****^	33.0 ± 14.25^****^	25.0 ± 0.98^****^
YZ	84.4 ± 5.63	60.9 ± 9.71^**^	64.6 ± 3.45^*^	48.6 ± 8.49^***^	46.2 ± 4.72^***^

####: p < 0.0001, versus Sham; *:p < 0.05, **:p < 0.01, ***:p < 0.001, ****:p < 0.0001,versus Model.

**FIGURE 2:**
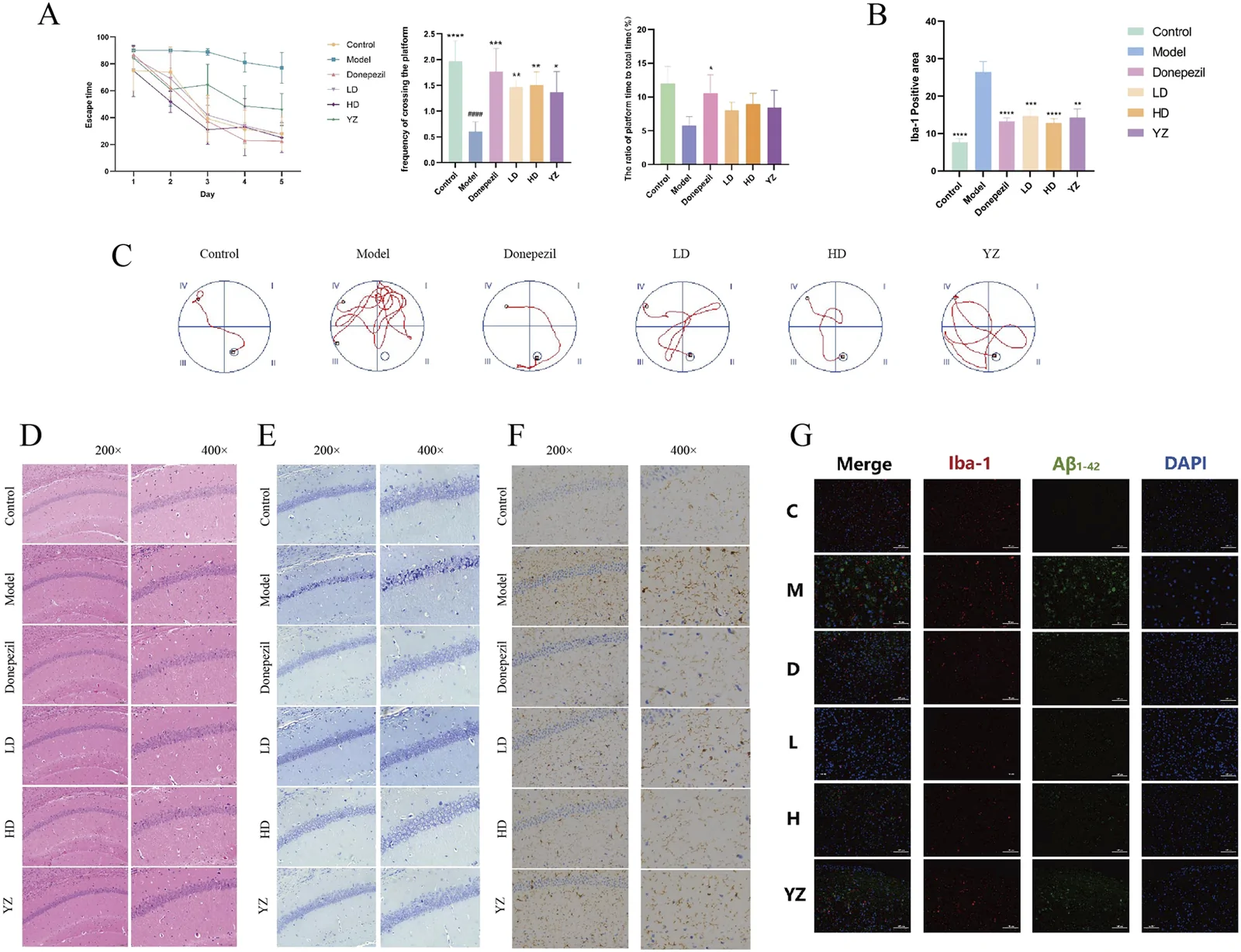
**(A)** Effects of Curcuma longa L. on Aβ_1-42_-induced spatial learning and memory in mice. **(B)** Effects of Curcuma longa L. on Aβ_1-42_-induced Iba-1 production (n = 3). Proportion of Iba-1 positive area in the hippocampus. **:p < 0.01,***:p < 0.001,****:p < 0.0001,versus Model. **(C)** Water maze route map. **(D)** Effects of Curcuma longa L. on Aβ_1-42_-induced pathological alterations (200× and 400×). **(E)** Curcuma longa L. reduces Aβ_1-42_-induced neuronal damage and loss (200×,400×). **(F)** Effects of Curcuma longa L. on Aβ_1-42_-induced Iba-1 production. IHC staining of Iba-1 in CA1 region (200×,400×). **(G)** Detection of Iba-1 and Aβ_1-42_ expression by IF staining (200×).

### 3.4 Curcumin and turmeric residue attenuate aβ_1-42_-induced neuronal damage in the hippocampal CA1 region

HE staining (Figure 2D) revealed well-organized neurons with clear nuclei in the hippocampal CA1 region of the sham group. In contrast, the model group exhibited significant neuronal damage, including cell deformation, pyknotic nuclei, and disordered arrangement, consistent with Aβ_1-42_-induced neurotoxicity (Zhang et al., 2013). All treatment groups showed a dose-dependent mitigation of these pathological changes, characterized by increased neuronal density, orderly arrangement, and restored nuclear clarity. The donepezil group exhibited the most pronounced improvement, followed by the high- and low-dose curcumin groups, and the *turmeric* residue group.

Nissl staining (Figure 2E) showed prominent nucleoli and abundant Nissl bodies in the sham group. The model group, however, displayed neuronal loss, absent nucleoli, and a marked reduction of Nissl bodies. Drug treatments ameliorated these abnormalities, leading to improved cellular integrity and increased Nissl body expression.

### 3.5 Curcumin and turmeric residue suppress aβ-induced microglial activation

The expression of Iba-1 in the hippocampal CA1 region was significantly elevated following Aβ_1-42_ injection, which was accompanied by an increase in Aβ_1-42_ deposition; both markers reached their highest levels in the model group. Under baseline conditions, only minimal Iba-1 expression was detected in the sham-operated group. As shown in Figure 2G, Iba-1 protein levels were significantly higher in the model group compared to the sham-operated controls. In contrast, all drug treatment groups exhibited a marked reduction in Iba-1 expression relative to the model group (Figures 2F,B).

### 3.6 Validation of the analytical stability

**FIGURE 3:**
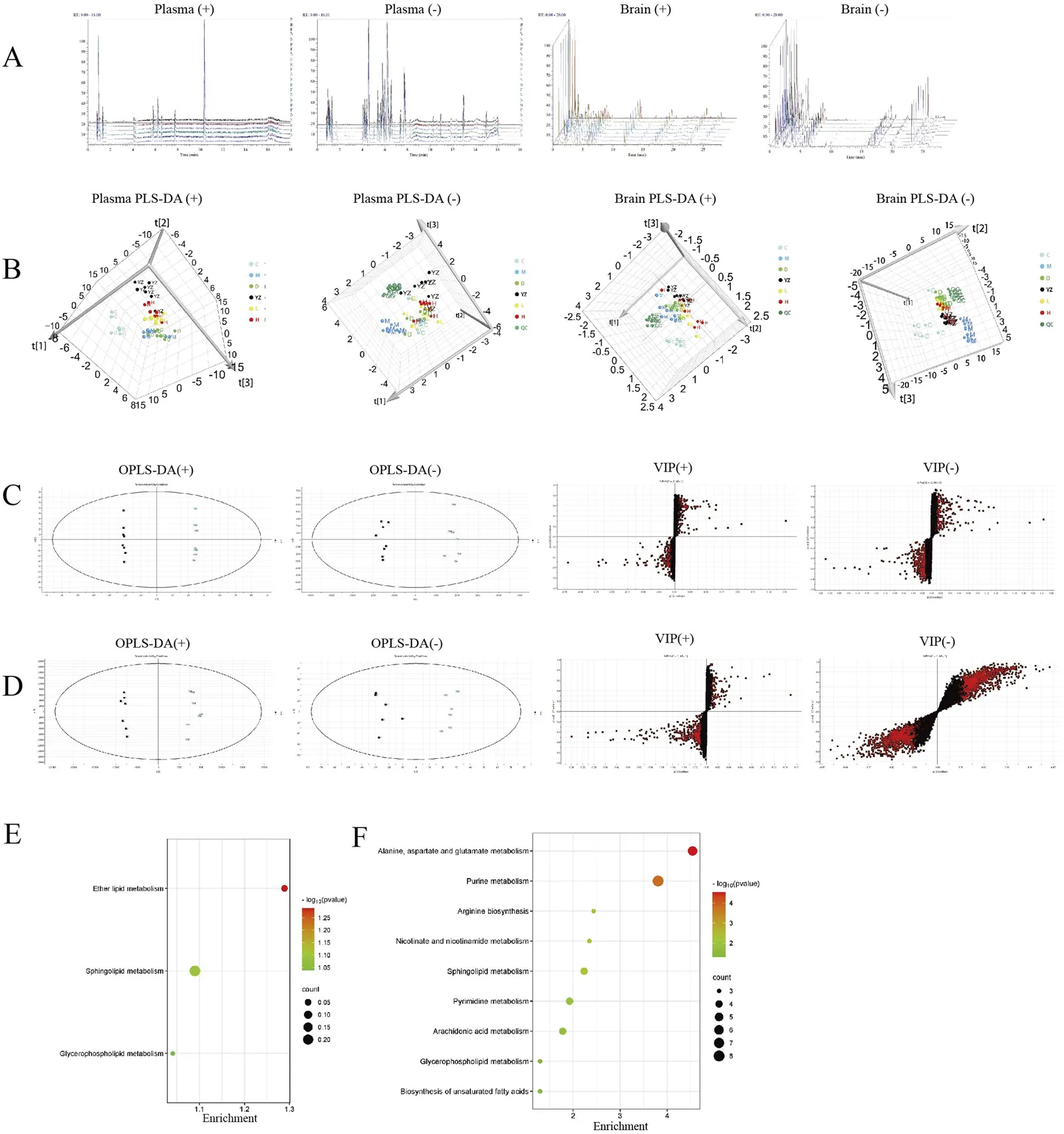
**(A)** Total ion chromatogram/ **(B)** Results of visualization of spatial distribution of untargeted metabolomics data. C: control group; M: model group; D: positive drug group; L: curcumin low-dose group; H: curcumin high-dose group; YZ: turmeric residue group. **(C)** The OPLS-DA and VIP plots of the plasma. **(D)** The OPLS-DA and VIP plots of the brain tissue. **(E)** KEGG metabolic pathway score map. **(F)** KEGG metabolic pathway score map.

The total ion chromatograms (TIC) of the quality control (QC) samples from both plasma and brain tissue in positive and negative ion modes were well-overlapped (Figure 3A), indicating satisfactory instrumental stability and reproducibility during the entire analysis. Furthermore, the stability was assessed using five representative endogenous metabolites. As summarized in Tables 3, 4, the relative standard deviations (RSD) of the peak areas for all analyzed metabolites were below 30%, confirming the reliability and robustness of the data for subsequent multivariate statistical analysis.

**TABLE 3:** Representative endogenous metabolite stability results.

Name	Formula	m/z	Rt [min]	Adduct	RSD Areas [%]
Palmitoylglycerophosphocholine	C24 H50 N O7 P	496.3396	6.2	[M + H]+1	14.1
Indoleacrylicacid	C11 H9 N O2	188.0707	4.1	[M + H]+1	6.7
Hexadecanamide	C16 H33 N O	256.2632	9.7	[M + H]+1	5.3
N-Acetylnorvaline	C7 H13 N O3	158.0811	4.1	[M-H]-1	21.0
Hydroxydodecanoicacid	C12 H24 O3	215.1646	5.5	[M-H]-1	14.1
Selenohomocysteine	C4 H9 N O2 Se	181.9730	4.6	[M-H]-1	10.4

**TABLE 4:** Representative endogenous metabolite stability results.

Name	Formula	m/z	RT (min)	Adduct	RSD Areas [%]
Trigonelline	C7 H7 N O2	138.0550	1.0	[M + H]+1	4.3
Sphingosine	C18 H35 N O2	298.2740	13.5	[M + H]+1	1.6
Phosphorylcholine	C5 H14 N O4 P	184.0733	17.1	[M + H]+1	3.5
Lactic acid	C3 H6 O3	89.02310	1.7	[M-H]-1	7.8
Guanosine monophosphate	C10 H14 N5 O8 P	362.0507	1.8	[M-H]-1	7.2
Guanosine	C10 H13 N5 O5	282.0841	3.6	[M-H]-1	8.0

### 3.7 Curcumin and turmeric residue ameliorate metabolic disorders in AD mice

Plasma (n > 6) and brain tissue samples (n > 6) in positive and negative ion modes are shown in PLS-DA models in Figure 3B. The model group and the control group can be distinguished, suggesting that there are significant changes in the body’s metabolism in the AD mice compared to normal mice. After the intervention of curcumin and *turmeric* dregs, the body’s metabolism of AD mice can be improved, showing a trend away from the model group and closer to the control group, indicating that the body’s metabolism of the demented mice after drug intervention tends to return to normal levels.

As shown in Figure 3B represent the PLS-DA score plots for plasma and brain tissue, respectively. As shown in Figures 3C,D represent the OPLS-DA and VIP plots for plasma and brain tissue, respectively.

The PLS-DA models constructed from plasma and brain tissue metabolomes (n > 6 per group) effectively discriminated the model group from the control group in both positive and negative ion modes (Figure 3B), indicating substantial metabolic disturbances in AD mice. Following intervention with curcumin and *turmeric* residue, the metabolic profiles of the treated groups shifted away from the model group and toward the control group in the PLS-DA score plots, suggesting a partial restoration of normal metabolic status.

### 3.8 Metabolomic analysis reveals the metabolic pathways underlying Curcumin’s amelioration of aβ-induced damage

In plasma, a total of 218 differential metabolites were screened (163 positive ions and 55 negative ions). Comparison and identification yielded 25 characteristic differential metabolites (21 positive ions and 4 negative ions), as shown in Table 5.

**TABLE 5:** Results of differential metabolite identification.

NO	Metabolite	Formula	m/z	RT (min)	Adduct
1	Allopurinol	C5 H4 N4 O	137.0459	4.1	[M + H]+1
2	2-Mercapto-2-methylpentan-4-one	C6 H12 O S	133.0682	4.2	[M + H]+1
3	(+)-bornane-2_5-dione	C10 H14 O2	167.1067	4.4	[M + H]+1
4	Mebeverine	C25 H35 N O5	430.2586	4.2	[M + H]+1
5	3-(Benzylsulfinyl)alanine	C10 H13 N O3 S	228.0689	4.3	[M + H]+1
6	3-Methylcatechol	C7 H8 O2	125.0597	4.1	[M + H]+1
7	Xanthine	C5 H4 N4 O2	153.0408	2.3	[M + H]+1
8	trans-2-Dodecenoylcarnitine	C19 H35 N O4	342.2638	4.6	[M + H]+1
9	[PK]Chrysophanol	C15 H10 O4	255.0650	4.3	[M + H]+1
10	2,3-Dimethoxy-5-methyl-6-(3-methyl-2-buten-1-yl)-1,4-benzenediol	C14 H20 O4	253.1433	5.1	[M + H]+1
11	(2E,4E,12Z)-N-Isobutyl-2,4,12-octadecatrienamide	C22 H39 N O	334.3106	12.6	[M + H]+1
12	Camphorsulfonic acid	C10 H16 O4 S	233.0842	4.7	[M + H]+1
13	Diphenamid	C16 H17 N O	240.1382	5.2	[M + H]+1
14	Chavicol	C9 H10 O	135.0803	4.9	[M + H]+1
15	4-[(1S)-1-cyclohexyl-2-(2-piperidinyl)ethyl]cyclohexanol	C19 H35 N O	294.2786	9.8	[M + H]+1
16	Dimethisterone	C23 H32 O2	339.2330	10.4	[M-H]-1
17	L-Threonic acid	C4 H8 O5	135.0298	1.3	[M-H]-1
18	Homovanillic acid sulfate	C9 H10 O7 S	261.0072	4.1	[M-H]-1
19	Guaiacol sulfate	C7 H8 O5 S	203.0011	4.1	[M-H]-1
20	n-Hexanamide	C6 H13 N O	115.0997	1.0	[M + H]+1
21	3-Methylcatechol	C7 H8 O2	124.0524	4.2	[M + H]+1
22	Oleamide	C18 H35 N O	282.2716	6.7	[M + H]+1
23	[FA (16:2)]N-hexadecyl-ethanolamine	C18 H37 N O2	299.2822	4.7	[M + H]+1
24	Sphinganine	C18 H39 N O2	301.2979	5.0	[M + H]+1
25	PC(18:3 (9Z,12Z,15Z)/18:2 (9Z,12Z))	C44 H78 N O8 P	779.5428	16.1	[M + H]+1

In brain tissue, a total of 428 differential metabolites were screened (307 positive ions and 121 negative ions). Comparison and identification yielded 38 characteristic differential metabolites (30 positive ions and 8 negative ions), as shown in Table 6.

**TABLE 6:** Results of differential metabolite identification.

NO	Metabolite	Formula	m/z	RT (min)	Adduct	Trend (M)/(C)	Trend (D)/(M)	Trend (CUR)/(M)	Trend (CHR)/(M)
1	Orthophosphate	H3 O4 P	98.9842	1.1	[M + H]+1	↓	↑	↑	↑
2	Indane	C9 H10	118.0782	14.6	[M + H]+1	↑	↓	↓	↓
3	Adenine	C5 H5 N5	135.05455	3.0	[M + H]+1	↑	↓	↓	↓
4	L-Threonic acid-1,4-lactone	C4 H6 O4	118.02802	3.0	[M + NH4]+1	↑	↓	↓	↓
5	L-Glutamic acid	C5 H9 N O4	147.05325	2.0	[M + H-H2O]+1	↑	↓	↓	↓
6	Cholinephosphate	C5 H14 N O4 P	183.0661	0.9	[M + H]+1	↑	↓	↓	↓
7	N-Undecanoylglycine	C13 H25 N O3	243.18338	24.5	[M + H]+1	↑	↓	↓	↓
8	Adenosine	C10 H13 N5 O4	267.09675	3.0	[M + H]+1	↑	↓	↓	↓
9	Linoleamide	C18 H33 N O	279.25601	21.1	[M + H]+1	↑	↓	↓	↓
10	Methyl palmitate	C17 H34 O2	287.28244	13.0	[M + H]+1	↑	↓	↓	↓
11	L-Glutathione oxidized	C20 H32 N6 O12 S2	612.1517	1.6	[M+2H]+2	↑	↓	↓	↓
12	15-Deoxy-Δ12,14-prostaglandin D2	C20 H30 O4	316.20383	12.8	[M + H]+1	↑	↓	↓	↓
13	15(R)-prostaglandin D2	C20 H32 O5	352.2251	10.8	[M + H]+1	↑	↓	↓	↓
14	Hypoxanthine	C5 H4 N4 O	136.03859	3.8	[M + H]+1	↑	↓	↓	↓
15	Bisphenol a diglycidyl ether	C21 H24 O4	357.19394	16.5	[M + H]+1	↑	↓	↓	↓
16	Xanthine	C5 H4 N4 O2	153.04085	3.9	[M + H]+1	↑	-	↓	↓
17	Guanine	C5 H5 N5 O	151.04945	3.4	[M + H]+1	↓	↑	↑	↑
18	Guanosine	C10 H13 N5 O5	283.09155	3.4	[M + H]+1	↑	↓	↓	↓
19	L-Glutamic acid	C5 H9 N O4	147.05325	2.0	[M + H-H2O]+1	↑	↓	↓	↓
20	L-pyroglutamic acid	C5 H7 N O3	129.04269	2.0	[M + H]+1	↑	↓	↓	↓
21	APM	C7 H13 N O4	175.08451	2.3	[M + H]+1	↑	↓	↓	↓
22	Sphinganine	C18 H39 N O2	301.2981	14.1	[M + H]+1	↑	↓	↓	↓
23	Oleic acid	C25 H43 N4 O5 P	510.29596	19.6	[M-H]-1	↓	↑	↑	↑
24	Pseudouridine	C9 H12 N2 O6	244.06951	2.1	[M + H]+1	↓	↑	↑	↑
25	Uracil	C4 H4 N2 O2	112.02733	2.1	[M + H]+1	↓	↑	↑	↑
26	Hannokinol	C19 H24 O4	316.16746	9.7	[M-H]-1	↓	↑	↑	↑
27	Prostaglandin D2	C20 H32 O5	352.2251	10.9	[M-H]-1	↑	↓	↓	↓
28	4-Undecylbenzenesulfonic acid	C17 H28 O3 S	312.17587	17.2	[M-H]-1	↓	↑	↑	↑
29	UDP-N-acetylglucosamine	C17 H27 N3 O17 P2	607.08134	1.0	[M-H]-1	↑	↓	↓	↓
30	Docosahexaenoic acid	C22 H32 O2	328.24019	22.8	[M-H]-1	↓	↑	↑	↑
31	Malonic acid	C3 H4 O4	104.01004	1.0	[M-H + HAc]-1	↓	↓	↓	↓
32	Glycolic acid	C2 H4 O3	76.01471	1.0	[M-H]-1	↑	↓	↓	↓
33	phenoxypropylpenicillin	C18 H22 N2 O5 S	378.12325	16.5	[M + H]+1	↑	↓	↓	↓
34	sn-glycero-3-Phosphoethanolamine	C5 H14 N O6 P	215.06316	0.909	[M + H]+1	-	-	-	-
35	Palmitoylcarnitine	C23 H45 N O4	400.34212	17.482	[M + H]+1	↓	↑	↑	↑
36	(2E)-hexadecenoylcarnitine	C23 H43 N O4	398.32635	16.104	[M + H]+1	↓	↑	-	↑
37	Acetyl-L-carnitine	C9 H17 N O4	204.12314	1.135	[M + H]+1	-	-	-	-
38	1-Palmitoylglycerophosphocholine	C24 H50 N O7 P	496.33986	16.619	[M + H]+1	-	-	-	-

↑ Indicated upregulation; ↓ Indicate downward adjustment; - Indicates uncontrol. C is the normal group; M is the module; D Donepezil group; CUR, is *turmeric* group; CHR, is *turmeric* residue group.

Based on the criteria of P < 0.05 and an effect size greater than 0, plasma exhibited enrichment in three potential metabolic pathways, as depicted in Figure 3E. Brain tissue demonstrated enrichment in eight potential metabolic pathways, as illustrated in Figure 3F. These findings suggest that curcumin may alleviate neuroinflammation induced by Aβ_1-42_ damage and improve memory and cognitive functions in AD patients through potential molecular pathways including ether lipid metabolism, sphingolipid metabolism, glycerophospholipid metabolism, alanine, aspartate, glutamate metabolism, purine metabolism, arginine biosynthesis, nicotinate and nicotinamide metabolism pyrimidine metabolism arachidonic acid metabolism.

## 4 Discussion

In the present study, we integrated chemical characterization, pharmacodynamic evaluation, spatial metabolomics, and untargeted metabolomics to investigate the neuroprotective effects of curcumin and turmeric residue in an Aβ_1-42_-induced AD mouse model. Our results demonstrated that both curcumin and turmeric residue significantly improved cognitive impairment, attenuated hippocampal pathological injury, suppressed microglial activation, and partially restored AD-associated metabolic disorders. These findings suggest that the therapeutic effects of turmeric and its residue are closely associated with the coordinated regulation of neuroinflammation and metabolic homeostasis.

Chemical characterization identified 132 compounds in turmeric and turmeric residue extracts, including curcumin, demethoxycurcumin, bisdemethoxycurcumin, tetrahydrocurcumin, cyclocurcumin, and multiple sesquiterpenoids. Previous studies have demonstrated that curcuminoids represent the principal bioactive constituents responsible for the antioxidant, anti-inflammatory, and neuroprotective activities of turmeric (Reddy et al., 2018). Among these compounds, tetrahydrocurcumin (THC), a major bioactive metabolite of curcumin, has attracted considerable attention due to its potent antioxidant and anti-inflammatory properties. Evidence suggests that THC alleviates neuronal injury through modulation of oxidative stress and neuroinflammatory signaling pathways. Mechanistically, THC has been shown to upregulate Gab2 and K-Ras expression while preventing the downregulation of Ccnd2, thereby attenuating G1/S phase arrest, suppressing aberrant microglial activation, and ultimately exerting neuroprotective effects in neurodegenerative disorders (Bhat et al., 2015). In addition to curcuminoids, emerging evidence indicates that other phytochemical constituents present in turmeric, including sesquiterpenoids and related volatile compounds, may also contribute to its anti-inflammatory and neuroprotective activities, suggesting that the therapeutic effects observed in the present study are likely mediated through the synergistic actions of multiple classes of bioactive compounds (Zhou et al., 2025). Furthermore, the chemical complexity of turmeric residue indicates that substantial amounts of biologically active constituents remain after industrial extraction, providing a plausible chemical basis for its observed pharmacological activity and supporting its potential for high-value reutilization as a functional medicinal resource (Dong et al., 2025).

Behavioral, histopathological, and immunohistochemical analyses consistently demonstrated that both curcumin and turmeric residue exerted significant neuroprotective effects in Aβ_1-42_-induced AD mice. HE and Nissl staining revealed substantial preservation of hippocampal neuronal morphology and integrity following treatment, while Iba-1 immunohistochemistry showed a marked reduction in microglial activation. Neuroinflammation is increasingly recognized as a central pathological driver of AD progression, linking amyloid deposition to neuronal dysfunction and cognitive decline. Accumulating evidence suggests that excessive Aβ accumulation triggers chronic activation of microglia, resulting in the release of pro-inflammatory cytokines, reactive oxygen species, and other neurotoxic mediators that exacerbate synaptic dysfunction and neuronal degeneration (Zhou et al., 2025). Consistent with this mechanism, lysophosphatidylcholine, a well-established pro-inflammatory lipid mediator, exhibited progressive accumulation in the hippocampal and cerebellar regions of model mice. Previous studies have demonstrated that lysophosphatidylcholine promotes the secretion of IL-6, IL-1β, IL-12, and TNF-α, thereby amplifying inflammatory responses and contributing to neuroinflammatory pathology (Guerreiro et al., 2013). Notably, both curcumin and turmeric residue significantly suppressed Iba-1 expression and partially reversed the abnormal accumulation of inflammation-associated metabolites, suggesting that attenuation of microglia-mediated neuroinflammatory responses may represent a key mechanism underlying their neuroprotective effects. Recent metabolomics studies have highlighted that inflammatory signaling and metabolic reprogramming are closely interconnected processes during neurodegenerative progression. These findings further support the concept that natural bioactive compounds can alleviate AD pathology through coordinated regulation of neuroinflammation and metabolic homeostasis, thereby preserving neuronal function and improving cognitive performance (Shi et al., 2025).

Spatial metabolomics and untargeted metabolomics analyses revealed extensive metabolic remodeling in Aβ_1-42_-induced AD mice, among which amino acid metabolism emerged as one of the most profoundly disturbed metabolic networks. Glutamate metabolism plays a central role in maintaining excitatory neurotransmission and neuronal homeostasis within the central nervous system. Accumulating evidence suggests that disruption of glutamate homeostasis contributes significantly to AD pathogenesis by promoting excitotoxicity through excessive activation of NMDA receptors, leading to intracellular calcium overload, mitochondrial dysfunction, oxidative stress, and ultimately neuronal degeneration (Menon and Sudheer, 2007). Consistent with this mechanism, the AD model mice exhibited marked alterations in glutamate-related metabolic pathways, accompanied by elevated levels of pyroglutamate and disturbances in arginine biosynthesis. Pyroglutamate has been reported to facilitate β-sheet formation and accelerate amyloid aggregation, thereby exacerbating neurodegenerative processes (Reinke and Gestwicki, 2007). Meanwhile, reduced argininosuccinate levels may reflect impaired arginine metabolism and dysregulated nitric oxide signaling, both of which have been implicated in neuronal injury and cognitive decline (Winter et al., 2015). Metabolomic evidence increasingly supports the notion that disruption of amino acid metabolism is closely associated with neuroinflammatory activation and disease progression in neurodegenerative disorders (Shi et al., 2025). These metabolic abnormalities collectively suggest that amino acid metabolic dysfunction may contribute to excitotoxicity, neuroinflammation, and progressive neuronal damage in AD. Importantly, treatment with curcumin and turmeric residue partially restored glutamate-related metabolic disturbances and normalized several amino acid metabolites toward physiological levels, indicating that regulation of amino acid homeostasis may represent an important mechanism underlying their neuroprotective effects. These findings support the growing view that metabolic reprogramming of neurotransmitter-associated pathways is closely linked to AD progression and that restoration of glutamatergic balance may contribute to the preservation of neuronal function and cognitive performance.

Purine and pyrimidine metabolism were also significantly disrupted in AD mice. Emerging evidence suggests that purine metabolites participate in multiple biological processes, including energy metabolism, neurotransmission, oxidative stress regulation, and neuroimmune signaling. Dysregulation of purine metabolism may contribute to mitochondrial dysfunction and neuronal energy deficiency, both of which are characteristic features of AD pathology. In the present study, the restoration of purine-related metabolic pathways following curcumin and turmeric residue treatment suggests an improvement in neuronal metabolic resilience and cellular energy homeostasis. Collectively, these observations support the emerging view that normalization of nucleotide metabolism may alleviate neurodegenerative processes and improve cognitive performance.

Lipid metabolism represented another major metabolic network affected in the AD model. Increasing evidence supports lipid metabolic remodeling as a critical pathological hallmark linking chronic inflammation, neuronal dysfunction, and neurodegeneration (Walsh et al., 2025). The observed alterations in sphingolipid and arachidonic acid metabolism are consistent with emerging evidence suggesting that inflammatory lipid mediators actively participate in neurodegenerative progression (Walsh et al., 2025). Significant alterations were observed in arachidonic acid metabolism, sphingolipid metabolism, and phospholipid turnover. Lipid metabolic dysfunction has increasingly been recognized as a hallmark of AD progression because lipids not only serve as structural components of neuronal membranes but also function as important signaling molecules involved in neuroinflammation and cell survival. Elevated levels of prostaglandin D_2_ suggest activation of inflammatory lipid signaling pathways, whereas alterations in sphingosine and phosphoethanolamine indicate disturbances in sphingolipid homeostasis. Accumulating evidence demonstrates that abnormal sphingolipid metabolism promotes ceramide accumulation, mitochondrial dysfunction, neuronal apoptosis, and microglial activation, thereby accelerating neurodegeneration. Mechanistic studies have revealed that dysregulated lipid metabolism actively participates in inflammatory signaling networks, establishing a pathogenic link between metabolic dysfunction, chronic inflammation, and progressive neurodegeneration (Walsh et al., 2025). The ability of curcumin and turmeric residue to normalize lipid metabolic disturbances therefore suggests that modulation of lipid-mediated inflammatory signaling may be a major mechanism responsible for their therapeutic effects.

Importantly, the metabolic pathways identified in this study are not isolated events but form an interconnected regulatory network associated with AD pathology. Amino acid metabolism, purine metabolism, and lipid metabolism collectively influence oxidative stress, neuroinflammation, mitochondrial function, neurotransmission, and neuronal survival. Previous studies have reported that curcumin interacts with multiple AD-related molecular targets, including inflammatory mediators, oxidative stress regulators, and signaling proteins involved in neuronal survival pathways. Recent studies on food–medicine homologous resources have emphasized that natural products often exert therapeutic effects through coordinated multi-component, multi-target, and multi-pathway regulation rather than through a single molecular mechanism (Ye et al., 2025). These targets are closely linked to the metabolic pathways identified in the present study. Taken together, these findings suggest that curcumin and turmeric residue exert multi-target and multi-pathway regulatory effects, ultimately leading to attenuation of neuroinflammation, restoration of metabolic homeostasis, and protection against neuronal injury.

An important finding of the present study is that turmeric residue retained considerable pharmacological activity despite being regarded as a by-product of industrial extraction. Both behavioral and metabolomic analyses demonstrated that turmeric residue produced therapeutic effects comparable to those of curcumin in several outcome measures. These findings suggest that substantial quantities of bioactive constituents remain in turmeric residue after extraction and support its potential as a valuable functional resource. In line with recent efforts aimed at the high-value utilization of medicinal and agricultural residues, our results provide experimental evidence supporting the sustainable reutilization of turmeric residue and highlight its potential economic and environmental significance (Li et al., 2025).

Several limitations of this study should be acknowledged. First, metabolite identification was primarily based on accurate mass measurements and MS/MS spectral matching and was not further confirmed using authentic reference standards. Second, although spatial metabolomics and untargeted metabolomics revealed significant metabolic alterations associated with curcumin and turmeric residue intervention, targeted validation of key metabolites was not performed. Third, while the present findings suggest that modulation of amino acid metabolism, purine metabolism, and lipid metabolism may contribute to the observed neuroprotective effects, direct molecular validation of the underlying signaling pathways was beyond the scope of this study. Future studies incorporating targeted metabolomics and molecular-level validation are warranted to further elucidate the mechanisms by which curcumin and turmeric residue exert therapeutic effects in AD. Such integrative approaches, combining metabolomics with systems-level analyses, have proven valuable for uncovering the neuroprotective mechanisms of natural products in AD models and may provide additional mechanistic insights in future investigations (Shaji et al., 2025). Beyond the contribution of small-molecule constituents, increasing attention has been directed toward plant-derived extracellular vesicles as important carriers of bioactive molecules. It would therefore be of considerable interest to investigate whether extracellular vesicles derived from turmeric residue contribute to the observed neuroprotective effects and participate in the regulation of brain metabolic networks. The integration of extracellular vesicle characterization with metabolomics and mass spectrometry imaging may provide new insights into the multi-component and multi-target therapeutic mechanisms of turmeric-derived products (Shu et al., 2025).

Collectively, the present findings indicate that curcumin and turmeric residue alleviate AD-associated pathological alterations through coordinated regulation of neuroinflammation and metabolic homeostasis. By restoring amino acid metabolism, purine metabolism, and lipid metabolism, these interventions may suppress microglial activation, reduce neuronal injury, and improve cognitive function. Furthermore, the discovery that turmeric residue retains significant neuroprotective activity highlights its potential as a novel functional resource and provides new perspectives for the high-value utilization of medicinal plant by-products.

## 5 Conclusion

This study systematically elucidated the neuroprotective effects and underlying mechanisms of curcumin and its residue extracts in Aβ_1-42_-induced AD mice by integrating air flow-assisted desorption electrospray ionization AFAI-MSI, untargeted metabolomics, and pharmacodynamic evaluation. Both curcumin and its residue significantly ameliorated spatial cognitive deficits, mitigated hippocampal neuronal damage, and attenuated neuroinflammation in AD mice. Using AFAI-MSI, we established a three-week post-Aβ_1-42_ injection as the optimal modeling period and delineated spatiotemporal dynamics of cerebral metabolic biomarkers. Metabolomics revealed that curcumin intervention notably restored dysregulated metabolic pathways, particularly sphingolipid and glycerophospholipid metabolism, in both brain tissue and plasma. Notably, mass spectrometry confirmed that curcumin residue retained core bioactive constituents (e.g., curcumin) while being enriched with differential metabolites such as turmeronols, which exhibit potential neuroprotective properties. These findings provide novel insights into AD metabolic pathology and support the translational potential of curcumin residue as a cost-effective AD therapeutic strategy. Future studies will focus on precise modulation of key metabolic targets and turmeronols’ neuroprotective mechanisms.

## Data Availability

The original contributions presented in the study are included in the article/supplementary material, further inquiries can be directed to the corresponding authors.
